# Feasibility of contrast-enhanced coronary artery magnetic resonance angiography using compressed sensing

**DOI:** 10.1186/s12968-020-0601-0

**Published:** 2020-02-13

**Authors:** Kuniaki Hirai, Teruhito Kido, Tomoyuki Kido, Ryo Ogawa, Yuki Tanabe, Masashi Nakamura, Naoto Kawaguchi, Akira Kurata, Kouki Watanabe, Osamu Yamaguchi, Michaela Schmidt, Christoph Forman, Teruhito Mochizuki

**Affiliations:** 1 Department of Radiology, Ehime University Graduate School of Medicine, Shitsukawa, Toon, Ehime 791-0295 Japan; 2 Department of Radiology, Saiseikai Matsuyama Hospital, 880-2, Yamanishi, Matsuyama, Ehime 791-8026 Japan; 3 Department of Cardiology, Saiseikai Matsuyama Hospital, 880-2, Yamanishi, Matsuyama, Ehime 791-8026 Japan; 4 Department of Cardiology, Ehime University Graduate School of Medicine, Shitsukawa, Toon, Ehime 791-0295 Japan; 5 Siemens Healthcare GmbH, Allee am Roethelheimpark 2, 91052 Erlangen, Germany

**Keywords:** Coronary magnetic resonance angiography, Compressed sensing, Cardiovascular magnetic resonance

## Abstract

**Background:**

Coronary magnetic resonance angiography (CMRA) is a promising technique for assessing the coronary arteries. However, a disadvantage of CMRA is the comparatively long acquisition time. Compressed sensing (CS) can considerably reduce the scan time. The aim of this study was to verify the feasibility of CS CMRA scanning during the waiting time between contrast injection and late gadolinium enhancement (LGE) scan in a clinical protocol.

**Methods:**

Fifty clinical patients underwent contrast-enhanced CS CMRA and conventional CMRA on a 3 T CMR scanner. After contrast injection, CS CMRA was scanned during the waiting time for LGE CMR. A conventional CMRA scan was performed after LGE CMR. We assessed acquisition times and coronary artery image quality for each segment on a 4-point scale. Visible vessel length, sharpness and diameter of right (RCA), left anterior descending (LAD), and left circumflex (LCX) coronary arteries were also quantitatively compared among the scans.

**Results:**

All CS CMRA scans were successfully performed within the LGE waiting time. The median total scan time was 207 s (163, 259 s) for CS and 785 s (698, 975 s) for conventional CMRA (*p* < 0.001). No significant differences were observed in image quality scores, vessel length measurements, sharpness, and diameter between CS and conventional CMRA.

**Conclusions:**

We could achieve all CS CMRA scans within the LGE waiting time. Contrast-enhanced CS CMRA could considerably shorten the scan time while maintaining image quality compared with conventional CMRA.

## Background

Cardiovascular magnetic resonance (CMR) is capable of comprehensive assessment of the heart including anatomy and function, myocardial tissue characteristics, and coronary artery without radiation exposure [1–4]. Coronary magnetic resonance angiography (CMRA) has been developed over the past three decades as a possible noninvasive alternative for visualizing coronary arteries [5]. The balanced steady-state-free-precession (bSSFP) sequence has achieved great success in CMRA imaging at 1.5 T because of its intrinsic high blood signal intensity and blood-myocardial contrast [6]. Contrast-enhanced whole-heart CMRA with segmented fast low-angle shot (FLASH) 3D spoiled gradient-echo sequence at 3 T also demonstrated better depiction of coronary segments compared with bSSFP coronary CMRA at 1.5 T [7]. The shortening of CMRA acquisition time has been made possible by novel multi-channel cardiac coils and high parallel imaging factors [8]. Nevertheless, a CMRA scan still requires a comparatively long acquisition time, and is affected by the issues of operator dependency and limited ease-of-use, which limit its general applicability in clinical practice.

The recent development of the mathematical theory of compressed sensing (CS) has also been applied to CMR [9]. CS is based on reconstructing an image from an incompletely filled k-space [10, 11]. The CS technique is known to reduce scan time considerably, and was recently applied to cine CMR [12–15]. Nakamura et al. reported that non-contrast CS CMRA could significantly shorten acquisition time, compared with conventional CMRA, in healthy subjects. In that study, almost all the CMRA scans were performed within 5 min [16].

In the clinical comprehensive CMR protocol, including cine CMR, perfusion CMR, late gadolinium enhancement (LGE) CMR, and CMRA, there is a waiting time of about 10 min after the injection of contrast medium for LGE CMR, and the CMRA scan is usually performed at the end of the protocol. We hypothesized that CMRA using CS could be routinely obtained within the LGE waiting time, thereby decreasing the total examination time and reducing patient burden. Therefore, the aim of this study was to verify the feasibility of CS CMRA scanning during the LGE waiting time in a clinical protocol.

## Materials and methods

### Study population

**Fig. 1:**
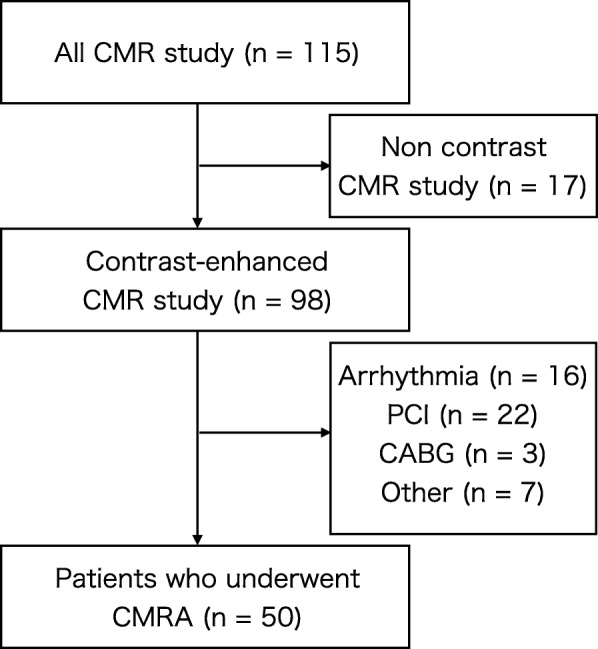
Flow diagram of patient recruitment. CMR: cardiovascular magnetic resonance, CMRA: coronary artery magnetic resonance angiography, PCI: percutaneous coronary intervention, CABG: coronary artery bypass graft

This is a prospective, two-center study. The study group consisted of 115 consecutive patients who were referred between August 2018 and March 2019 to undergo CMR for suspected cardiac disease. Exclusion criteria included non-contrast study, arrhythmia, patients with coronary stents or bypass grafts, and contraindications to CMR (claustrophobia, pacemaker). Sixty-five patients were excluded for these reasons, and 50 patients underwent CMRA (Fig. 1). The characteristics of the 50 patients are shown in Table 1. The institutional review board approved the study, and all participants gave written informed consent.

**Table 1:** Characteristics of study population

Number	50
Age (y)	60.5 ± 16.0
Sex (female/male)	21/29
Height (cm)	162.0 ± 9.0
Weight (kg)	59.1 ± 12.1
BMI (kg/m^2^)	22.4 ± 3.9
Hypertension	20 (40%)
Dyslipidemia	14 (28%)
Diabetes mellitus	13 (26%)
Smoking	24 (48%)
Family history of CAD	17 (34%)
Ischemia/Non ischemia	14/36

The data are presented as the mean ± standard deviation or as the number (%) of subjects

*BMI* Body mass index; *CAD* coronary artery disease

### CMR protocol

**Fig. 2:**
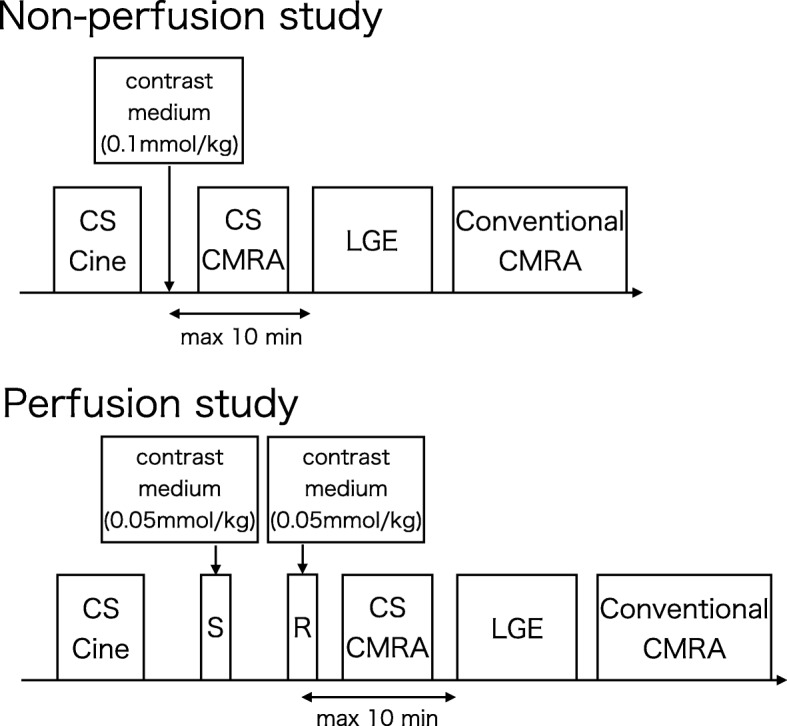
Study protocol. CS: compressed sensing, CMRA: coronary magnetic resonance angiography, LGE: late gadolinium enhancement, S: Stress perfusion, R: Rest perfusion

CMR images were acquired using a 3 T whole-body CMR system (MAGNETOM Skyra; Siemens Healthineers, Erlangen, Germany). The CMR protocol typically included CS cine function, LGE, and CMRA (Fig. 2). Following the acquisition of CS cine imaging, gadobutrol (0.1 mmol/kg) was injected intravenously at a rate of 0.5 mL/s with saline flash. LGE CMR was performed 10 min after the injection of contrast medium. CS CMRA, including preparatory steps such as detection of the data acquisition window and administration of nitroglycerin, was performed during the waiting time between contrast injection and LGE scan [17, 18], and conventional CMRA was performed after LGE CMR. If myocardial perfusion study was needed, gadobutrol (0.05 mmol/kg) was injected intravenously at a rate of 4 mL/s with saline flash for each stress and rest perfusion imaging (total 0.1 mmol/kg). LGE CMR was performed 10 min after the last contrast injection for rest perfusion imaging, and CS and conventional CMRA scans were performed as described above. If CS CMRA, including the preparatory steps, could not be completed within 10 min after contrast injection, the CS CMRA scan was terminated and considered failed, and the LGE scan was immediately performed.

### CMRA

CMRA was obtained using electrocardiography (ECG)-triggered and navigator-gated techniques. CMRA scans were obtained using a T2-prepared segmented 3D spoiled gradient echo sequence [19]. Conventional CMRA was compared to a CS prototype sequence. Imaging parameters are shown in Table 2. A nitroglycerin sublingual spray (0.3 mg) was administered before each CMRA scan. A patient-specific data acquisition window length was determined in a 4-chamber cine sequence during either systole or diastole, according to the minimal motion of the right coronary artery (RCA) [20]. Respiratory motion was detected by placing a cross-pair navigator on the dome of the right hemi-diaphragm and data was only acquired in the expiratory phase. Respiratory acceptance rate was defined as the ratio of the acquired data within the setting window.

**Table 2:** Image parameters

	CS CMRA	Conventional CMRA
Sequence type	spoiled gradient echo	spoiled gradient echo
TR/TE (ms)	3.2/1.4	3.2/1.4
FOV (mm)	320 × 258	320 × 258
Matrix	272 × 220	272 × 220
Actual voxel size (mm)	1.2 × 1.2 × 1.8	1.2 × 1.2 × 1.8
Reconstruction voxel size (mm)	1.2 × 1.2 × 0.9	0.6 × 0.6 × 0.9
Acquisition window and Number of profiles acquired per heartbeat	adapted to the individual heart rate of the subject	same as possible for CS imaging
Bandwidth (Hz/pixel)	593	613
Acceleration factor	7.6	2 (GRAPPA)
Acceptance window	± 3.0 mm	± 3.0 mm

*CS* compressed sensing; *FOV* field of view; *GRAPPA* generalized autocalibrating partially parallel acquisitions; *TE* echo time; *TR* repetition time

### Data acquisition and image reconstruction CS CMRA

Sparse incoherent sampling of the Cartesian phase-encoding plane was performed with a variable-density spiral phyllotaxis pattern for data acquisition [21]. The acceleration factor was set to 7.6 compared with the fully sampled k-space. After data acquisition, images were reconstructed using CS reconstruction with a 3D spatial regularization using redundant Haar wavelets as described in [22]:

$$ \underset{\mathbf{x}}{\min }{\left\Vert \mathbf{Ax}-\mathbf{y}\right\Vert}_2^2+\lambda {\left\Vert \phi \left(\boldsymbol{x}\right)\right\Vert}_1, $$

where the first part of the equation optimizes the data fidelity of the estimated volume **x** with the acquired data **y**. **A** represents the system matrix containing the Fourier transform, coil sensitivity maps, and sampling pattern, and **x** the estimated image. The second term enforces a sparse representation of the image using a l1-norm regularization after the wavelet transformation *ϕ*(). This cost function was optimized with a modified fast iterative shrinkage thresholding algorithm (FISTA) [23]. The second term was weighted with the regularization parameter λ, which was set to 0.0035 for both the phase-encoding and the slice-encoding direction regularization. For all datasets, the iterative reconstruction was terminated after 20 iterations.

### Qualitative image quality analysis

Two radiologists with 10 years (reader 1) and 4 years (reader 2) of CMR experience independently graded image quality based on a four-point scale with respect to the border definition of coronary arteries, as follows: 1) severely blurred; 2) markedly blurred; 3) mildly blurred; 4) sharply defined. The artifact score was also graded with respect to wrap around, respiratory ghost, and cardiac ghost as follows: 1) severe artifacts; 2) some artifacts; 3) few artifacts; 4) no artifacts. Coronary segments were defined according to the classification of the American Heart Association [24], as follows: #1: RCA proximal; #2: RCA middle; #: RCA distal; #5: left anterior descending (LAD) main; #6: LAD proximal; #7: LAD middle; #11: left circumflex (LCX) proximal; and #13: LCX distal. Image quality and artifacts were assessed in the axial orientation. Image quality was evaluated for each segment and artifacts for the entire image. We used the image quality assessed by reader 1 and computed the inter-observer agreement with reader 2 for subsequent analyses.

### Quantitative vessel analysis

CS and conventional CMRA images were transferred to an external workstation (SYNAPSE VINCENT, Fujifilm Corp., Ltd., Tokyo, Japan). A third radiologist, with 8 years of CMR experience and blinded to the origin of the images, performed curved planar reconstruction (CPR) of RCA, LAD, and LCX from each set of axial CMRA images, and measured the length of the vessels on CPR images semi-automatically using the workstation.

The sharpness and diameter of the vessels were evaluated on RCA#1, LAD#6, and LCX#11. Signal intensity profiles along a user-defined line perpendicular to the major axes of the vessel were obtained. Vessel diameter was calculated as the full width at half between maximum and background [25]. To evaluate the sharpness of the vessel, the 20 and 80% points between the maximal and background signal intensities were first calculated for each side of the signal intensity profile. The distance in millimeters between the two points was then determined for each side. Vessel sharpness was defined as the inverse of the averaged distance of the two sides [26].

### Statistical analysis

Continuous variables are presented as mean ± standard deviation (SD) or as median (first quartile, third quartile). Acceptance rates, heart rates, sharpness, and diameter were compared between CS and conventional CMRA using paired t-tests. Scan times, image quality scores, artifact scores, and visible vessel length were compared between the two methods using the Wilcoxon matched-pairs signed-rank test. Correlation and agreement between the visible vessel lengths were assessed using linear regression and Bland-Altman analysis. The quadratic-weighted kappa test was used to evaluate the inter-observer agreement of image quality. A *P*-value (*p*) < 0.05 was considered as statistically significant, and Bonferroni correction was used to reduce the chance of obtaining false-positive results (type I errors) when multiple pair wise tests were performed in the quantitative vessel assessments (e.g., visible vessel length, sharpness, and diameter). Statistical analysis was carried out using the statistical software (JMP version 13; SAS Institute, Cary, North Carolina, USA).

## Results

All CS and conventional CMRA scans were successfully performed for all 50 patients. The LCX proximal and distal segments (#11, #13) of one patient showed hypoplasia and were excluded from the analysis. In total, 149 vessels and 348 coronary artery segments from 50 patients were evaluated. The median total scan time, including navigator efficiency, was 207 s (163, 259 s) for CS and 785 s (698, 975 s) for conventional CMRA (*p* < 0.001). The respiratory acceptance rate was 49.7 ± 9.5% for CS CMRA, and 51.4 ± 8.7% for conventional CMRA (*p* = 0.077). The heart rate was 66.5 ± 11.4 bpm for CS CMRA and 67.2 ± 12.8 bpm for conventional CMRA (*p* = 0.79). CS reconstruction was performed inline at the scanner at the end of the acquisition using graphical processing units (GPUs) on the MR reconstruction system. We could reconstruct the CS CMRA images in approximately 2 min.

### Qualitative image quality analysis

**Fig. 3:**
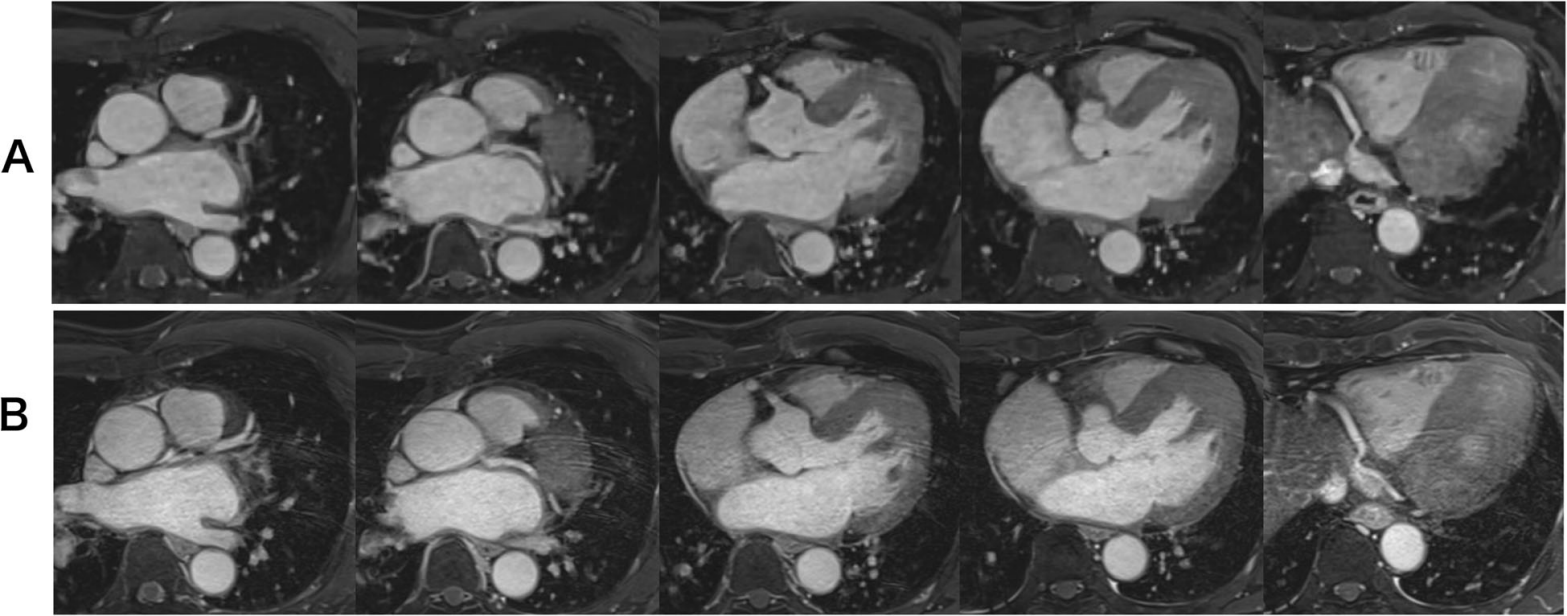
Axial images of CS (**a**) and conventional (**b**) CMRA. Both image sets were acquired from a 63-year-old patient. Both observers rated the image quality as excellent (4 points) for both techniques. CS largely shortened the acquisition time (2 min 6 s for CS, 10 min 0 s for conventional)

**Fig. 4:**
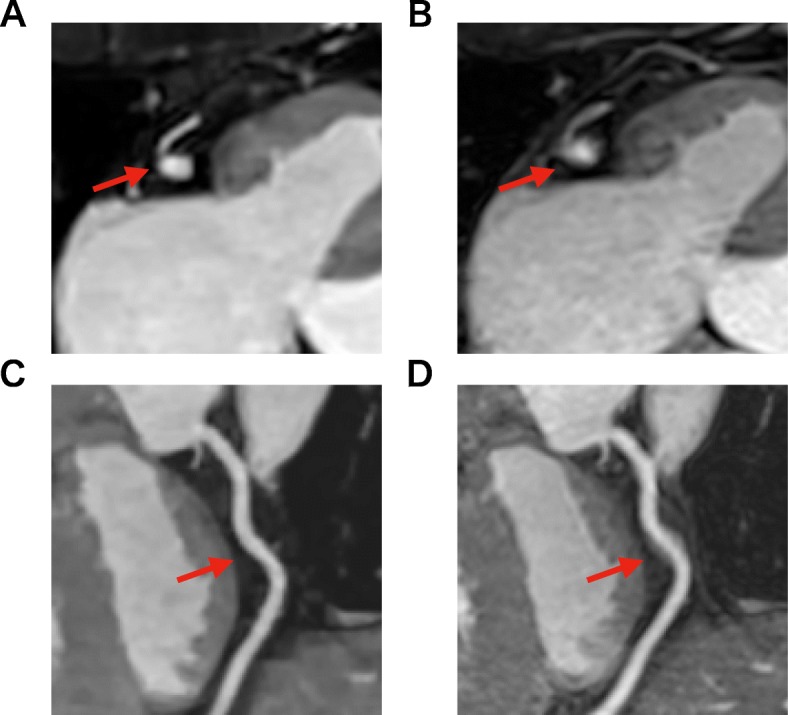
Axial images and curved planar reconstruction for the right coronary artery (RCA) in the two techniques. Both image sets were acquired from a 65-year-old patient. Conventional CMRA had motion artifacts on RCA (**b**, **d**), while CS CMRA did not (**a**, **c**). Both observers rated the image quality for RCA mid as good (3 points) in conventional imaging (**b**) and excellent (4 points) in CS imaging (**a**)

Table 3 summarizes the image quality scores in each coronary artery segment. No significant differences were found in image quality scores and artifact scores between CS and conventional CMRA. The two radiologists showed good agreement in subjective image quality scores (Kappa = 0.72 for CS CMRA, Kappa = 0.74 for conventional CMRA) and artifact scores (Kappa = 0.79 for CS CMRA, Kappa = 0.77 for conventional CMRA). Figures 3 and 4 shows examples of CS and conventional CMRA for image analysis.

**Table 3:** Image quality scores

	CS CMRA	Conventional CMRA	*p*-value
RCA proximal	3.6 ± 0.7	3.5 ± 0.7	0.110
RCA mid	3.5 ± 0.8	3.6 ± 0.7	0.411
RCA distal	3.5 ± 0.7	3.5 ± 0.6	0.622
LAD proximal	3.7 ± 0.5	3.6 ± 0.5	0.376
LAD mid	3.6 ± 0.8	3.5 ± 0.7	0.316
LAD distal	3.3 ± 0.8	3.2 ± 0.8	0.600
LCX proximal	3.5 ± 0.6	3.5 ± 0.6	0.444
LCX distal	3.2 ± 0.9	3.3 ± 0.7	0.145
Artifact	3.8 ± 0.4	3.8 ± 0.4	0.730

The data are presented as the mean ± standard deviation

*CS* compressed sensing; *LAD* left anterior descending artery; *LCX* left circumflex artery; *mid* middle; *RCA* right coronary artery

### Quantitative vessel analysis

**Fig. 5:**
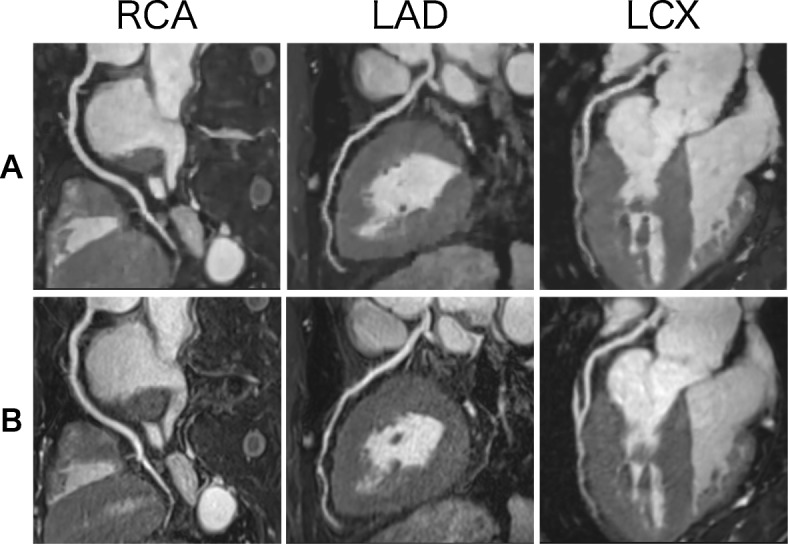
Curved planar reconstruction of CS (**a**) and conventional (**b**) CMRA. Both image sets were acquired from a 63-year-old patient. Visible vessel length was similar in the two techniques (RCA: CS 154 mm vs. conventional 156 mm; left anterior descending (LAD): CS 148 mm vs. conventional 149 mm; left circumflex (LCX): CS 127 mm vs. conventional 129 mm)

**Fig. 6:**
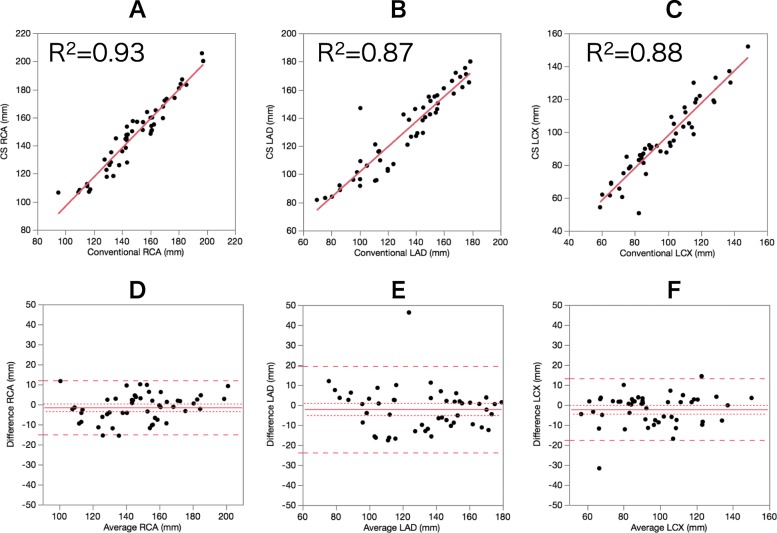
Scatter plots and Bland-Altman plots for the visible vessel length in the two techniques. In Bland-Altman plots, the solid line indicates the mean difference between the two techniques; the long-dashed lines indicate the corresponding double standard deviation intervals; and the short-dashed lines indicate the 95% confidence interval of the mean difference. **a**/**d**: right coronary artery, **b**/**e**: left anterior descending artery, **c**/**f**: left circumflex artery

Table 4 summarizes the quantitative assessment of each coronary artery. No significant differences were observed in vessel length, sharpness, and diameter between CS and conventional CMRA. Representative CPR images of the two methods for the evaluation of visible vessel length are shown in Fig. 5. There was good agreement between CS and conventional CMRA for all measurements (Fig. 6).

**Table 4:** Quantitative vessel assessment

	CS CMRA	Conventional CMRA	*p*-value
Vessel length (mm)			
RCA	149 (128, 161)	146 (132, 163)	0.189
LAD	134 (103, 155)	138 (110, 155)	0.079
LCX	91 (80, 111)	93 (82, 114)	0.153
Vessel sharpness (1/mm)			
RCA	0.87 ± 0.14	0.85 ± 0.16	0.152
LAD	0.90 ± 0.15	0.87 ± 0.14	0.090
LCX	0.95 ± 0.19	0.94 ± 0.16	0.301
Vessel diameter (mm)			
RCA	4.0 ± 0.6	4.1 ± 0.6	0.514
LAD	3.6 ± 0.6	3.5 ± 0.6	0.492
LCX	3.2 ± 0.5	3.2 ± 0.5	0.701

The data are presented as the mean ± standard deviation or as the median (first quartile, third quartile)

*CS* compressed sensing; *LAD* left anterior descending artery; *LCX* left circumflex artery; *RCA* right coronary artery

## Discussion

From this prospective, two-center study, we make the first report on the feasibility of contrast-enhanced CS CMRA in a clinical protocol. CS CMRA enabled a substantial reduction in acquisition time while maintaining image quality compared with conventional CMRA. Due to the short acquisition time, all CS CMRA scans were successfully performed within the waiting time (10 min) for LGE in the clinical protocol.

CMRA has emerged as a method capable of providing visualization of coronary arteries without ionizing radiation [27, 28]. Shorter acquisition times have become possible using bSSFP sequences, parallel imaging with multi-channel coils, respiratory gating with navigator echoes, and improved strategies for k-space sampling [29, 30]. Today, the major problems with CMRA are the relatively long acquisition time and image artifacts caused by motion instability during the scan. Recently, new rapid imaging methods such as self-navigation and CS have emerged which can overcome these problems [18, 31–33].

In our study, CS CMRA considerably shortened the acquisition time compared with conventional CMRA in clinical patients. In the clinical protocol, LGE CMR is commonly obtained 10 min after injection of contrast medium [34]. Our results showed that the examination time of CS CMRA, including preparatory steps such as the detection of the data acquisition window and the administration of nitroglycerin, is short enough to allow the scan to be performed within the waiting time between contrast injection and LGE scan.

The acquisition time of CS CMRA was very short and the acquisition data was smaller than conventional CMRA; therefore, the image quality of CS CMRA might be expected to be poorer. While a previous 3 T non-contrast study [16] showed inferior results from CS CMRA in terms of image quality and visible vessel length compared with conventional CMRA, our study did not reveal significant differences in either aspect. Furthermore, we found no significant difference in the quantitative evaluation of image quality as defined by sharpness. Several reasons were considered as to why the qualitative and quantitative evaluations with CS and conventional CMRA were not significantly different. Since conventional CMRA is performed at the end of the examination, it may be influenced by the patient’s movement due to fatigue from the long CMR examination. In our study, CS CMRA could be performed at an early stage, thus decreasing patient fatigue and movement. The motion of the heart through the cardiac and respiratory cycles can also cause blurring and artifacts in CMR studies [35]. The short acquisition time of CS CMRA may also reduce the artifacts produced by unstable respiratory conditions and motion during the scanning (Fig. 4). Respiratory self-navigation has been proposed as an alternative approach to compensate for respiratory motion while also increasing scan efficiency [18, 31–33]. Further shortening of the imaging time can be expected by the combination of CS and self-navigation.

Differences in contrast agent concentration in the coronary artery at the time of imaging should also be considered. Contrast medium improves the signal-to-noise and contrast-to-noise ratios in CMRA, with an emphasis on the role of T1-shortening [36]. In our protocol, CS CMRA scans were performed immediately after injection of the contrast medium when the concentration of contrast agent was high. In comparison, since conventional CMRA was performed after LGE, the contrast agent had washed out over time and its concentration was relatively lower. Double-dose contrast medium (0.2 mmol/kg) is widely used for LGE [37]. However, in our study, single-dose (0.1 mmol/kg) medium was used because double-dose is not permitted in Japan. The single-dose was likely to reduce its concentration over time faster than the double-dose, leading to an advantage for CS CMRA that could be performed immediately after the injection of the contrast medium. In the CS technique, the contrast in the image plays a major part in the ability to reconstruct vastly undersampled images. High contrast often results in large distinct sparse coefficients [9]. These reasons may help to explain the lack of significant differences between CS and conventional CMRA for qualitative and quantitative evaluations. Contrast-enhanced CS CMRA could shorten the acquisition time considerably while maintaining image quality compared with conventional CMRA.

In this study, we verified that adequate CS CMRA could be performed during the waiting time in a normal clinical protocol. Preparatory steps such as the detection of the data acquisition window and the administration of nitroglycerin were performed after contrast medium administration so as not to disturb the clinical examination. If preparatory steps are performed before contrast medium injection, it can maximize the blood gadolinium concentration during CS CMRA acquisition and allow more time for CS CMRA scanning.

In addition, CS can also potentially improve spatial resolution [38]. In our results, CS CMRA scan times were significantly shorter than the 10-min limit imposed by the LGE waiting time. Therefore, by spending more time on the CS CMRA acquisition, image quality can be improved, or isotropic resolution can be achieved.

In the future, further high resolution CMRA imaging may allow for detailed coronary artery assessment. We can expect such new protocols to replace conventional CMRA with CS CMRA performed during the waiting time. This protocol will significantly shorten the total CMR examination time and lead to a reduction in patient burden.

### Limitations

**Fig. 7:**
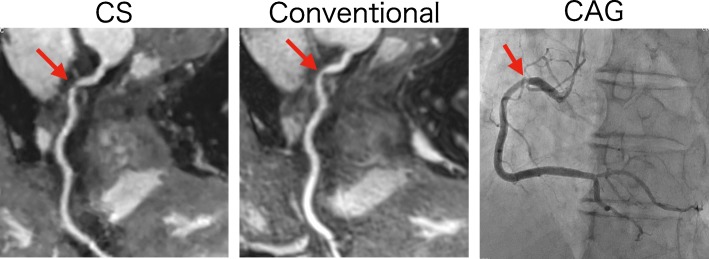
A 59-year-old patient with chest pain. Significant coronary artery stenosis in the RCA is observed in the CS (**a**) and conventional (**b**) CMRA, with good correlation with coronary angiography (**c**)

Our study has several limitations. First, we evaluated only 50 patients and did not perform stenotic vessel evaluations. A previous study of contrast-enhanced conventional CMRA with a 3 T scanner showed detection of significant coronary artery disease (CAD) with good diagnostic performance [8]. We did not evaluate stenosis because coronary angiography was performed in only a few cases. However, in the quantitative evaluation of the diameter, there was no significant difference between CS and conventional CMRA, suggesting that CS had little effect on the diameter. Furthermore, we could present a representative case with significant CAD detected in CS CMRA as well as in conventional CMRA (Fig. 7). We expect that further studies will reveal the accuracy of CS CMRA in assessing significant CAD.

Second, complete blinding in the evaluation of image quality was not achieved. When we visually evaluated the images, we could readily distinguish CS coronary images from conventional coronary images, because CS CMRA shows less noise and has a slightly pixelated and textured appearance compared with conventional CMRA. This factor may have contributed to bias in the evaluation of coronary image quality.

Third, in our protocol, all CS CMRA scans were performed first, followed by conventional CMRA scans; random sequence ordering was not performed. This may have been advantageous for CS CMRA due to the higher concentration of contrast agent and less patient movement from fatigue. As CS and conventional CMRA could not be compared under the same conditions, there may be a bias. However, if the conventional CMRA scan is performed first, the time to LGE scan after contrast medium ejection will be longer, which will decrease the contrast concentration and lead to a reduction in LGE image quality in clinical practice [39]. Since the acquisition time is short, CS CMRA examination can be performed in the waiting time without deteriorating LGE imaging.

Fourth, there were few cases of obesity within our Japanese patient cohort. When CMRA examination is performed with a Western population where body mass index is often much higher in patients suspected of CAD, the image quality may be decreased due to poor fat saturation and aliasing [40]. Larger fields of view also require longer acquisition time assuming the image resolution is unchanged. Therefore, our results may be not always applied to a Western patient population. In addition, the abdominal belt technique, which suppresses breathing-related motion of the diaphragm, was not used in the current study so as to not disturb the clinical examination. The use of an abdominal belt is expected to further improve image quality and shorten acquisition time [5, 41].

## Conclusions

The acquisition time of CS CMRA is short enough to allow the scan to be performed within the waiting time for LGE, thereby shortening the protocol time while maintaining image quality as compared with conventional CMRA performed after LGE imaging. These findings suggest the possibility of a faster CMR protocol.

## Supplementary information

**Additional file 1.** Histogram distribution of the image quality scores

## Data Availability

Not applicable.

## Abbreviations

bSSFP: Balanced steady state free precession
CAD: Coronary artery disease
CMR: Cardiovascular magnetic resonance
CMRA: Coronary magnetic resonance angiography
CPR: Curved planar reconstruction
CS: Compressed sensing
ECG: Electrocardiogram
FISTA: Fast iterative shrinkage threshold algorithm
FLASH: Fast low-angle shot
GPU: Graphical processing unit
LAD: Left anterior descending coronary artery
LCX: Left circumflex coronary artery
LGE: Late gadolinium enhancement
RCA: Right coronary artery
