# Supplementary material for: Feasibility of contrast-enhanced coronary artery magnetic resonance angiography using compressed sensing
Source: J Cardiovasc Magn Reson. 2020 Feb 13;22:15. doi: 10.1186/s12968-020-0601-0 (PMC7017458; doi:10.1186/s12968-020-0601-0)
Supplement: Supplementary file 1 — Additional file 1. Histogram distribution of the image quality scores [file 12968_2020_601_MOESM1_ESM.pdf]

Histogram distribution of the image quality scores

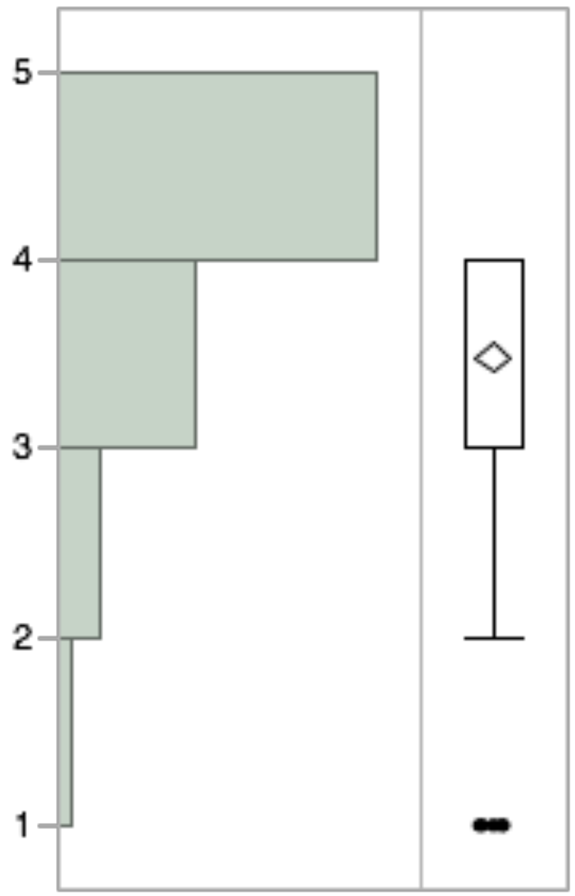

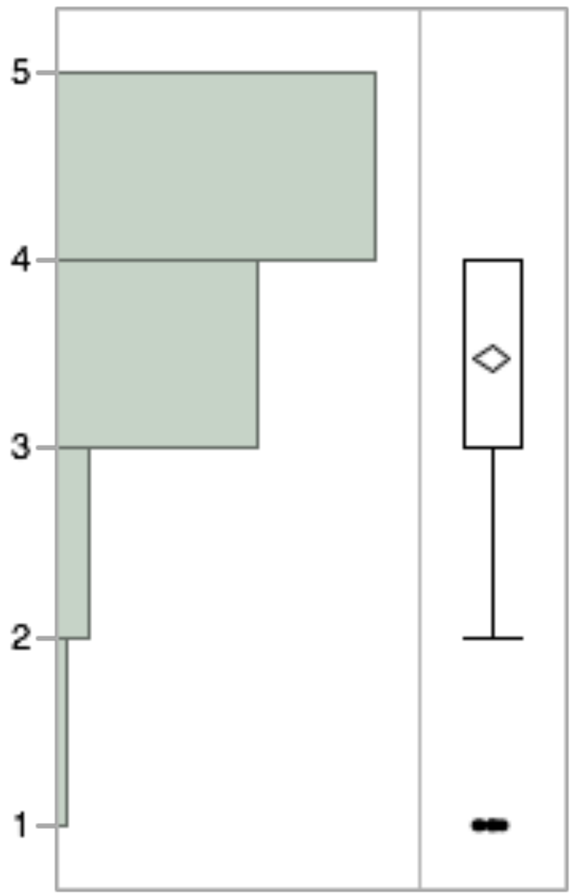

image quality score	CS (number of segment)	Conventional (number of segment)
4	248	227
3	107	143
2	32	23
1	11	5
